# No Decision About Me Without Me: International Views on Providing Care Against Someone’s Will

**DOI:** 10.1093/geroni/igaa057.2298

**Published:** 2020-12-16

**Authors:** Michel Bleijlevens, Jan Hamers, Elizabeth Capezuti

## Abstract

People with cognitive impairment may experience the care provided by their caregiver(s) as unnecessary or undesirable, which is expressed by behaviors such as resisting the efforts of a caregiver or preventing the caregiver to perform or assist with ADL such as bathing, dressing and toileting. This can lead to stress, agitation and aggression for both the care recipient and the caregiver, and places the caregiver in a complex dilemma. Should the caregiver force hygiene or respect the person’s autonomy to refuse care? It is difficult for caregivers to find a balance between quality of care and safety, while accepting the person’s autonomy. Caregivers in long-term care often feel the necessity to provide care against the will of people with a cognitive impairment, including physical restraints, psychotropic medication, and non-consensual care. This symposium provides an international perspective with presenters from Belgium, Switzerland, Germany, and the Netherlands. The first presenter explores the use and factors associated with physical restraint, psychotropic medication, and non-consensual care in people with dementia receiving home care in the Netherlands and Belgium. Second, a presenter from Switzerland focuses on the prevalence of restraint use in nursing homes. The third presenter introduces an Advance Care Planning intervention from Germany that aims to ensure that personal wishes in home care are followed. The last presenter assesses the feasibility of an approach to prevent and reduce care against a person’s will in the Netherlands. To conclude, our discussant will integrate these insights and draw conclusion for policy, practice and further research. Systems Research in Long-Term Care Interest Group Sponsored Symposium

